# Insights into Cross-Kingdom Plant Pathogenic Bacteria

**DOI:** 10.3390/genes2040980

**Published:** 2011-11-28

**Authors:** Morgan W.B. Kirzinger, Geetanchaly Nadarasah, John Stavrinides

**Affiliations:** Department of Biology, University of Regina, 3737 Wascana Parkway, Regina, Saskatchewan S4S0A2, Canada; E-Mails: morgankirzinger@gmail.com (M.W.B.K.); gnadarasah@gmail.com (G.N.)

**Keywords:** cross-kingdom, human pathogens, host specificity, multi-host pathogens, reservoirs, evolution, insects

## Abstract

Plant and human pathogens have evolved disease factors to successfully exploit their respective hosts. Phytopathogens utilize specific determinants that help to breach reinforced cell walls and manipulate plant physiology to facilitate the disease process, while human pathogens use determinants for exploiting mammalian physiology and overcoming highly developed adaptive immune responses. Emerging research, however, has highlighted the ability of seemingly dedicated human pathogens to cause plant disease, and specialized plant pathogens to cause human disease. Such microbes represent interesting systems for studying the evolution of cross-kingdom pathogenicity, and the benefits and tradeoffs of exploiting multiple hosts with drastically different morphologies and physiologies. This review will explore cross-kingdom pathogenicity, where plants and humans are common hosts. We illustrate that while cross-kingdom pathogenicity appears to be maintained, the directionality of host association (plant to human, or human to plant) is difficult to determine. Cross-kingdom human pathogens, and their potential plant reservoirs, have important implications for the emergence of infectious diseases.

## 1. Introduction

Plant pathogenic bacteria are responsible for major economical losses in agricultural industries worldwide, prompting massive research efforts to understand their ecology, pathology and epidemiology. Studies of many agriculturally relevant pathogens like *Pseudomonas syringae*, *Xylella fastidiosa*, *Erwinia amylovora*, and *Xanthomonas campestris* [1-4] have revealed an extensive specialization to plant systems, including a wealth of plant-specific pathogenicity determinants such as type III secretion systems, plant hormone analogs, and enzymes that target plant-specific cell wall components [1,5-7]. Despite this, we often neglect the fact that the interactions of phytopathogenic bacteria are not confined to plants, and may include other organisms in the environment. Recent studies have begun to show that many plant pathogens have the capacity to colonize other hosts outside of the plant kingdom, including insects, animals, and humans [8,9].

Human pathogens are studied almost exclusively for their detrimental impact on human health. Pathogens such as *Escherichia coli*, *Listeria monocytogenes*, and *Campylobacter jejuni* possess a diverse set of genetic factors that enable pathogenicity, ranging from specialized secretion systems to toxins and adhesins, all of which are involved in manipulating or circumventing the human immune system [10-12]. We often consider these human pathogenic bacteria to be dedicated animal pathogens, causing disease and epidemics; yet, the constant interaction of human carriers with their environment predisposes these pathogens to alternative niches that include non-animal hosts. Not surprisingly, we have begun to discover that some animal pathogens, which are known to cause serious human diseases, also have plant pathogenic capabilities. The epidemiology and disease strategies of these pathogens, whether considered primarily a plant or human pathogen, are of particular interest from the perspectives of both the biology and evolution of cross-kingdom pathogenesis.

The disease strategies used by cross-kingdom pathogens to infect unrelated hosts are of particular interest since plant and animal hosts have distinctive physical barriers and defense responses. While specialists may have evolved dedicated factors for overcoming the physical barriers and innate defenses in a certain host, a cross-kingdom pathogen would require a diverse library of genes and disease strategies to overcome the specific defense obstacles of each of its hosts. This would either necessitate a suite of pathogenicity factors to enable attachment, disease development, and dispersal for each host, or a universal disease strategy in which the same suite of pathogenicity factors is used for all hosts. Signatures of host adaptation will be apparent in these determinants, which may include incremental mutations and genetic rearrangements, along with the acquisition of novel genetic elements that may contribute to virulence or host specificity [13]. The identification of these genetic determinants in cross-kingdom pathogens with both human- and plant-pathogenic potential can provide a better understanding of the evolution of phytopathogenicity, as well as the role of plants as potential reservoirs for clinically relevant bacteria.

This review will examine several bacterial pathogens that exhibit cross-kingdom pathogenicity, where both humans and plants are potential hosts (Table 1). We begin by exploring the pathogenicity of *Pantoea* and *Burkholderia*, which are commonly regarded as plant pathogens, but have been shown to cause human disease. We then examine the disease strategies of the human pathogens *Salmonella*, *Serratia*, *Enterobacter* and *Enterococcus*, which have been shown to have plant-pathogenic potential. We highlight what is known about the disease determinants and strategies for each pathogen in each of its hosts, and identify those examples where specific determinants are used in multiple hosts. Finally, we address the evolutionary means by which plant pathogens may evolve to be pathogenic to humans, as well as the possible routes of microbes that can lead to the evolution and maintenance of cross-kingdom pathogenicity.

## 2. Plant Pathogens that Infect Humans

Plant pathogens have evolved a repertoire of pathogenicity factors that are used to invade plant host cells, facilitate disease development, and eventually promote pathogen dispersal [14]. Surprisingly, some of these plant pathogens cause disease in humans, and are frequently isolated from human infections in the nosocomial environment. The genus *Pantoea*, for example, currently includes seven species (*Pantoea agglomerans*, *Pantoea ananatis*, *Pantoea citrea*, *Pantoea dispersa*, *Pantoea punctata*, *Pantoea stewartii*, and *Pantoea terra*) [15-21] that are known to cause plant disease. *P. agglomerans* causes crown and root gall disease of gypsophila and beet [15,20], *P. ananatis* causes bacterial blight and dieback of Eucalyptus [16], brown stalk rot of maize [22], and stem necrosis of rice [23], and *P. citrea* is the causal agent of pink disease in pineapple [21]. The type III secretion system (T3SS) appears to be an essential plant pathogenicity factor for many species of *Pantoea*, enabling efficient colonization and onset of disease through the injection of bacterial effector proteins that disrupt defense signalling in host cells [24]. *Pantoea agglomerans* pvs. *gypsophilae* and *betae* are known for using a T3SS for causing galls on their host plants [25]. While *P. agglomerans* pv. *betae* can induce gall formation on gypsophila and beet plants, *P. agglomerans* pv. *gypsophilae* is only capable of causing gall formation on gypsophila. Host range is determined in part by the type III effector protein, PthG, which is recognized by the beet plant resulting in a defense response, but functions as a virulence factor in gypsophila [25]. Studies with transgenic *Nicotiana tabacum* plants expressing PthG suggest that the effector may interfere in the plant auxin signalling pathways, resulting in higher observed auxin and ethylene levels, and subsequent blockage of root and shoot development [25]. The manipulation of plant-specific hormones is likely directly responsible for callus/gall formation, and highlights the specialization of this pathogen to plant systems.

Despite this specialization to plants, species of *Pantoea* have also been discovered to be pathogenic to humans. Now classified as an opportunistic human pathogen, *P. agglomerans* was implicated in US and Canadian outbreaks of septicaemia caused by contaminated closures on infusion fluid bottles [26]. *P. agglomerans* has since been associated in the contamination of intravenous fluid, parenteral nutrition, blood products, propofol, and transference tubes, causing illness and even death [27-31]. *P. agglomerans* has also been obtained from joint fluids of patients with synovitis, osteomyelitis, and arthritis [17], where infection often occurs following injuries with wood slivers, plant thorns, or wooden splinters [17,32-34]. This is also true for *P. ananatis*, *P. septica*, and *P. dispersa*, which are known for causing disease in onion and sugar cane plants, but have been implicated in multiple cases of bacteremia and septicaemia [35-37]. Phylogenetic studies examining the relationship between plant and clinical isolates have shown that they are indistinguishable, making their potential pathogenicity in plant and animal hosts unclear [38].

Much like *Pantoea*, species of *Burkholderia* are also recognized phytopathogens, but are ubiquitous in the terrestrial and aquatic environment. *Burkholderia* species, including *Burkholderia cepacia*, *Burkholderia cenocepacia*, *Burkholderia ambifaria*, and *Burkholderia pyrrocinia* were initially identified as inhabitants of agricultural soil, with some, like *Burkholderia glathei* being found in fossil soils in Germany [39]. Given their ubiquity in the terrestrial environment, it is not surprising that some strains, like *Burkholderia plantarii* and *Burkholderia gladioli* have been shown to be pathogenic to onion, rice, gladiolus and iris [39], and possess key plant pathogenicity factors. *Burkholderia glumae*, the most important bacterial pathogen of rice in Japan, Korea and Taiwan, was shown to carry a plant T3SS, which is essential for its ability to cause plant disease [40]. An analysis of the proteins regulated by HrpB, which activates the expression of T3SS genes, identified 34 secreted extracellular proteins, 21 of which had putative HrpB-binding sequences in their upstream regulatory regions [41]. Another set of 16 proteins appeared to be secreted via a type II protein secretion system (T2SS), and may play a role in plant pathogenesis [41]. Mutants lacking either the T2SS or the T3SS produced the toxin, toxoflavin, but were less virulent to rice panicles, indicating the importance of these systems to disease [41]. In fact, *Burkholderia pseudomallei* possesses multiple T3SS gene clusters, one of which, T3SS2, showed high similarity to the T3SSs of phytopathogenic *Xanthomonas* spp. and *Ralstonia solanacearum* [42], and in addition, arabinose-negative strains of *B. pseudomallei* are more virulent in tomato plants, and tend to carry a T3SS, suggesting that these two phenotypes are linked to virulence in plants [43]. Strains of some species, like *B. cepacia*, are specialized to plant roots and have been found in the maize rhizosphere [44], while others are pathogenic to onion, and cause severe rots using a cocktail of plant-specific enzymes following biofilm formation [45].

Interestingly, *B. cepacia* that causes onion rot can also cause life-threatening pulmonary infections in individuals with chronic granulomatous disease, or cystic fibrosis (CF) [46]. Between 1980 and 1990, *B. cepacia* was associated with a number of cases of “cepacia syndrome” in CF treatment centres and social gatherings, where CF patients became infected by other *B. cepacia*-carrying individuals, with many of the infections being fatal [39,47-50]. Recently, fatal infections were also observed in non-CF patients in reanimation wards in Europe and North America [51]. Investigation into the ability of various strains of *B. cepacia* to penetrate airway barriers revealed that all three of the *B. cepacia* strains tested circumvented the mechanical barriers of mucus and ciliary transport to penetrate the airway epithelium [52]. Different strains of *B. cepacia* use distinct invasion pathways and virulence determinants, which may account for differences in the virulence of strains [52].

Several disease determinants for different species of *Burkholderia* in human hosts have been determined. For example, *B. pseudomallei*, which infects tomato plants, contains a complete *pqsA-pqsE* operon, which is highly similar to the genes responsible for the synthesis of the virulence-associated signalling molecule 2-heptyl-3-hydroxy-4(1H)-quinolone (PQS) found in *Pseudomonas aeruginosa* [53]. Introduction of the *B. pseudomallei hhqA* and *hhqE* genes into *P. aeruginosa pqsA* and *pqsE* mutants restored PQS production and virulence. Likewise, the presence of a capsular polysaccharide has been implicated in the pathogenicity of *Burkholderia* toward humans. Parental strains of *B. mallei* with the capsule were highly virulent in hamsters and mice, while capsule mutants were avirulent in both animal models [54].

**Table 1:** Summary of cross-kingdom pathogens.

**Pathogen**	**Plant host/Niche**	**Plant pathogenicity factors** 1	**Human disease/Condition**	**Human pathogenicity factors** 1
*Enterobacter cloacae* 2	Macadamia, dragon fruit, orchids, papaya	Unknown	Respiratory/skin/urinary infection, septicaemia	cytotoxin, *ompX*
*Enterococcus faecalis* 2	*Arabidopsis thaliana*	*fsrB, sprE*	Urinary/abdominal/cutaneous infections, septicaemia	Hemolysin, *salB, esp, fsrB, sprE*
*Burkholderia ambifaria*	Soil, maize roots	Dioxygenase	Unknown	Unknown
*Burkholderia cenocepacia* 2	Soil, maize roots, onion	T4SS	Septicaemia	Unknown
*Burkholderia cepacia* 2	Soil, rice, maize, wheat, onion	Unknown	Septicaemia	Unknown
*Burkholderia gladioli* 2	Onion, gladiolus, iris, rice	Unknown	Septicaemia	Unknown
*Burkholderia glathei*	Soil	Unknown	Unknown	Unknown
*Burkholderia glumae* 2	Rice	*tox*R, T3SS	Chronic granulomatous disease	Unknown
*Burkholderia mallei* 2	Soil	Unknown	Melioidosis/Glanders	Capsular polysaccharide
*Burkholderia plantarii* 2	Rice, gladiolus, iris	*rpoS*, quorum sensing	Melioidosis	Rhamnolipids
*Burkholderia pseudomallei* 2	Tomato	T3SS	Melioidosis/Glanders	*pqsA–pqsE* operon
*Burkholderia pyrrocinia* 2	Soil	Unknown	Melioidosis/Glanders	Unknown
*Pantoea agglomerans* 2	Crown/root gall	*pthG*, T3SS	Arthritis/septicaemia	T3SS
*Pantoea ananatis* 2	Eucalyptus, maize, rice	T3SS	Septicaemia	Unknown
*Pantoea citrea* 2	Pineapple	T3SS	Septicaemia	Unknown
*Pantoea dispersa* 2	Seeds	T3SS	Septicaemia	Unknown
*Pantoea punctata*	Japanese mandarin oranges	T3SS	Unknown	Unknown
*Pantoea septica*	Unknown	Unknown	Septicaemia	Unknown
*Pantoea stewartii*	Maize	T3SS	Unknown	Unknown
*Pantoea terrea*	Japanese mandarin oranges	Unknown	Unknown	Unknown
*Salmonella enterica* 2	Tomato, *Arabidopsis thaliana*	*agfA, agfB,* FilI	Gastroenteritis/typhoid fever	Flagellum (*fhD*), T3SS
*Serratia marcescens* 2	Squash, pumpkin	Fimbrial genes, biofilm, *oxyR*	Septicaemia, urinary tract infection	LPS, iron uptake, hemolysin, protease

1 T3SS: type III secretion system; T4SS: type IV secretion system;

2 Bacterial pathogens capable of both plant and human disease.

## 3. Human Pathogens that Can Infect Plants

Our human-centric view of disease often leads to sweeping assumptions that human pathogenic bacteria are devoted to human hosts. Species of *Salmonella*, *Serratia*, *Enterobacter*, and *Enterococcus* are commonly considered problematic human pathogens that are frequently found in the nosocomial environment, and which cause food poisoning, general infections, and septicaemia [55-58] (Table 1). Relatively recent studies, however, have begun to uncover that these human-pathogenic bacterial species are also capable of colonizing and causing disease in a wide variety of plant hosts. Notably, many of these studies have been conducted under laboratory conditions, providing evidence for the phytopathogenic potential of these bacterial species; but, the incidence of plant disease caused by many of these human pathogens in the natural environment remains unknown.

*Salmonella* is the causal agent of many human, animal, and bird diseases worldwide, including gastroenteritis and typhoid fever, and results in approximately 1.4 million human illnesses and 600 deaths annually in the United States [56]. Successful host infection by *Salmonella* appears dependent on many pathogenicity determinants, including two T3SSs and a large suite of secreted effectors that function to mediate both intercellular and intracellular survival in the host [59]. Other virulence factors include the *flhD* gene, which regulates the production of the flagellum, is essential for the full invasive potential of the bacterium [60]. Virulence factors like flavohemoglobin protect against nitric oxides, and mediate bacterial survival in macrophages after phagocytosis [61].

Despite its clear adaptation to surviving in human hosts, *Salmonella* has also been isolated from the phyllosphere of tomato crops [62]. *S. enterica* levels on wild tomato (*Solanum pimpinellifolium*) were lower than on domesticated tomato cultivars (*Solanum lycopersicum*), with S. *enterica* preferentially colonizing type 1 trichomes. Plants irrigated with contaminated water had larger S. *enterica* populations than plants grown from seeds planted in infected soil; however, both routes of contamination resulted in detectable *S. enterica* populations in the phyllosphere, suggesting that *S. enterica* has the ability to associate with plants and has adapted to survive in the phyllosphere. *agfA* and *agfB* were identified as being involved in enabling *S. enterica* to associate with plants [63]. *agfA* mutants were unaffected in their ability to attach or colonize alfalfa sprouts, whereas *agfB* mutants showed reduced colonization ability. This suggests that *agfB* alone plays a role in plant attachment [63].

*Salmonella* is not only capable of colonizing the phyllosphere; it has been shown to infect the plant *Arabidopsis* under laboratory conditions, and cause death of plant organs [64,65]. Inoculation of *Salmonella* in *Arabidopsis* via shoot or root tissues resulted in chlorosis, wilting, and eventually death of the infected tissues within seven days [64]. The specific virulence factors involved are not yet known; however, its plant pathogenicity, much like its human pathogenicity appears dependent, in part, on the flagellum. FliI, a protein needed for flagellar assembly has been shown to be essential for plant pathogenesis, possibly through its involvement in a specialized protein export pathway [66]. The FliI protein is similar to the HrpB6 protein of the rice pathogen *Xanthomonas*, which is a key component of the T3SS, and mediates interactions between *Xanthomonas* and its host [66]. Secretion of proteins may therefore be integral for both human and plant association for *Salmonella*, although it is still unknown whether the same proteins can be used for pathogenesis in both hosts.

*Serratia marcescens* is a human pathogen commonly found in the respiratory and urinary tracts of humans, and is responsible for approximately 1.4% of nosocomial infections in the United States [57]. Through transposon mutagenesis, genes involved in lipopolysaccharide (LPS) biosynthesis, iron uptake, and hemolysin production were discovered to be essential for bacterial virulence in the host *Caenorhabditis elegans* [67]. Further studies have shown that purified protease proteins of *S. marcescens* administered to the lung tissue of guinea pigs and mice produced pneumonia-like symptoms and haemorrhaging, which was similar to those animals with acute *Serratia* pneumonia [68]. Yet, despite its animal virulence factors, *Serratia* has also been found to be a common phytopathogen. *S. marcescens* is recognized as a phloem-resident pathogen that causes cucurbit yellow vine disease of pumpkin (*Cucurbita moschata* L.) and squash (*Cucurbita pepo* L.), which is marked by wilting, phloem discoloration, and yellowing of foliage [69-71]. Recent studies have shown that *S. marcescens* produces a biofilm along the sides of the phloem vessels, blocking the transport of nutrients and eventually causing the plant to wilt and die [72,73]. A genetic screen to identify genes that modulate biofilm formation in *S. marcescens* revealed the involvement of fimbrial genes, as well as an *oxyR* homolog—a conserved bacterial transcription factor having a primary role in the oxidative stress response [74]. The involvement of these plant disease genes in human pathogenicity has not been determined.

Another bacterium that has also shown cross-kingdom pathogenesis is the Gram-negative bacterium *Enterobacter cloacae*, an important nosocomial pathogen responsible for bacteremia, lower respiratory tract infections, skin and soft-tissue infections, as well as urinary tract infections [55]. Recently, an *E. cloacae* infection of the bloodstream was traced back to contaminated human albumin [75]. *E. cloacae* synthesizes a Shiga-like toxin II-related cytotoxin, which was implicated in an infant case of haemolytic-uremic syndrome [76]. Studies have shown that the concentration of the outer membrane protein (OmpX) produced by *E. cloacae* during infection influenced the ability of the bacterium to invade rabbit intestinal tissue [77]. Overproduction of OmpX led to a 10-fold increase in the invasiveness of *E. cloacae* in rabbit intestinal enterocytes *in situ*, whereas the mutant and wildtype strains were unable to effectively invade the same tissue [77]. Interestingly, OmpX shares high amino acid similarity to the virulence proteins PagC and Rck of *Salmonella typhimurium* as well as the virulence-associated Ail protein of *Yersinia enterocolitica.* Although there is evidence that *E. cloacae* has evolved to colonize the human host, it has also been identified as the causal agent of grey kernel disease of macadamia (*Macadamia integrifolia*) [78]. The onset of grey kernel disease affects not only the quality of the kernels produced by the tree, but results in grey discoloration and a foul odour [78]. *E. cloacae* also causes bacterial soft rot disease in dragon fruit (*Hylocereus* spp.) [18], bacterial leaf rot in *Odontioda* orchids [79], and is also responsible for internal yellowing disease in papaya [78]. Again, the specificity of the virulence factors used in each of these hosts is not known.

Cross-kingdom pathogenesis is not limited to Gram-negative bacteria. Enterococci are part of the normal intestinal flora of humans and animals, but are also important pathogens responsible for serious infections, especially in immunocompromised patients [58]. With increasing antibiotic resistance, enterococci are recognized as nosocomial pathogens that can be challenging to treat. The genus *Enterococcus* includes more than 17 species, but only a few can cause local or systemic clinical infections including urinary tract and abdominal infections, wound infections, bacteremia, and endocarditis [80]. Clinical isolates of *Enterococcus faecalis* have been found to produce hemolysin—a virulence factor that leads to the lysis of red blood cells [81]. Hemolytic strains exhibit multiple drug resistance more frequently than non-hemolytic strains, while strains isolated from fecal specimens of healthy individuals display a low (17%) incidence of hemolysin production [81]. The *salB* gene of *E. faecalis* has also been shown to be a virulence factor, since it increases bacterial adherence to extracellular matrix proteins and promotes biofilm formation during infection [82]. The surface protein Esp was shown to contribute to the colonization and persistence of the bacterium in the urinary tract [83]. *E. faecalis* has also been shown to exhibit pathogenicity toward insects in addition to human hosts, where the extracellular gelatinase of *E. faecalis* was found to destroy the host defence system through the degradation of inducible antimicrobial peptides in insect hemolymph and in human serum [84]. Similarly, a putative quorum-sensing system gene (*fsrB*) and a serine protease (*sprE*) were shown to play an important role in mammalian and nematode models of infection [58].

*E. faecalis* is not only capable of infecting mammalian and nematode hosts, but also the plant *Arabidopsis thaliana*, causing plant death seven days after inoculation under laboratory conditions [58]. The manifestation of disease initially begins once the bacterium has successfully attached itself to the leaf surface, where upon entry of the leaf tissue through the stomata or wounds, *E. faecalis* multiplies and colonizes the intercellular spaces of the plant host and causes rotting and disruption of the plant cell wall and membrane structures. The phytopathogenicity of *E. faecalis* appears to involve some of the same genetic determinants involved in animal pathogenesis, including the quorum sensing system gene (*fsrB*) and a serine protease (*sprE*) [58]. The quorum-sensing mutant (Δ*fsrB*) was attenuated in virulence in the *A. thaliana* root model, since fewer bacteria were able to attach to the root surfaces, and biofilm formation was greatly reduced. Similarly, the serine protease mutant (Δ*sprE*) no longer caused plant death, suggesting that this gene plays an important role in *E. faecalis* plant pathogenesis [58]. *E. faecalis* clearly uses a general disease strategy that allows it to use the same virulence factors for exploiting two different hosts.

## 4. Evolutionary Models

**Figure 1:**
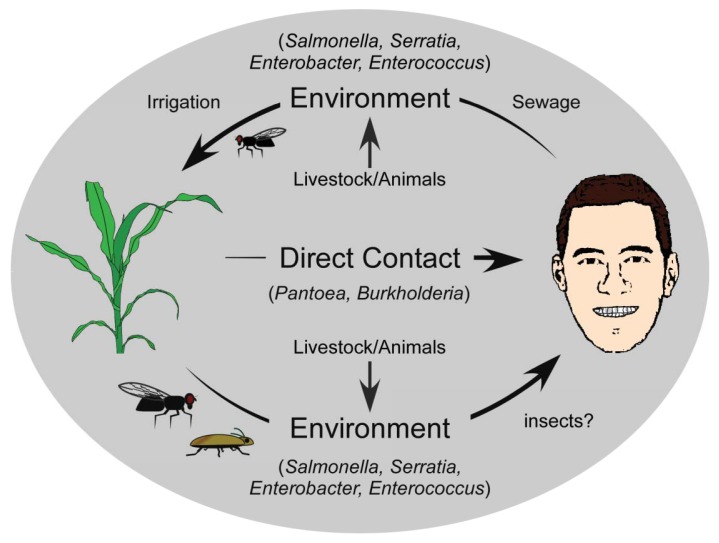
Cycling of cross-kingdom pathogens between plants and humans. Movement of human-associated bacteria (*Salmonella*, *Serratia*, *Enterobacter*, *Enterococcus*) into the general environment may occur through wastewater, with additional microbial input coming from agricultural and livestock runoff. Irrigation using contaminated water, along with vectoring by insects can lead to inoculation of plant surfaces or plant soils. Movement of potential human pathogenic bacteria from plants is also likely facilitated by insects, which can disperse bacteria into the general environment. The route back to humans is less clear, although some pathogens like *Burkholderia* and *Pantoea* can cause direct infections following cutaneous lesions or injuries from thorns or splinters.

Cross-kingdom pathogenicity represents an intriguing balance between the costs and benefits of bacterial generalization *versus* specialization. While there are benefits to specializing on one host, and evolving to exploit its subtle vulnerabilities, the availability of this host would limit the success of the pathogen, thereby favouring host diversification. Conversely, a broad host range would ensure host availability for the pathogen, but at the significant cost of having to utilize a universal, and likely suboptimal infection strategy for exploiting that host. When the alternative host is from a different kingdom, the dynamics become even more intriguing, since the disease strategy is likely to be quite different. The evolution of cross-kingdom pathogenicity and its origins can be described using several simplistic models. In the context of the examples described here, one model describes the evolution of a successful animal pathogen that acquires the ability to cause disease in a plant host (human to plant). If we presuppose that animal pathogens are readily acquired from the environment and are already adapted to animal hosts [85], there are several ways in which these pathogens may acquire plant-pathogenic potential. Deposition of animal pathogens back into the environment, such that they are introduced near or onto plants recurrently may facilitate the exchange of genetic information with other organisms in the environment, and the gain of a selective advantage that enables persistence. One primary route to this general environment is through the natural strategies used by bacteria to ensure dissemination from their infected host. For humans, symptoms such as diarrhea that result from an infection provide the bacteria with an escape route from the host into the environment [86] (Figure 1). Released bacteria move from human wastes to natural watersheds, with subsequent reuse of these sources for irrigation of commercially relevant crops increasing bacterial titres in the phyllosphere [64,65]. But, the movement of human pathogens to the general environment may also occur through indirect routes. The Pharoah ant (*Monomorium pharaonis*) is able to transport human pathogenic bacteria including *Salmonella*, *Staphylococcus*, and *Streptococcus* within a nosocomial setting [87], and there have been reports of the common house fly, *Musca domestica*, carrying *Pseudomonas aeruginosa*, *Enterococcus faecalis* and *Staphylococcus aureus* [88] (Figure 1). These insects function as transports for human pathogenic bacteria, moving them from the clinical setting to the general environment [9], and likely onto plants. Acclimation to a plant host would occur over extended periods of time, and would be accelerated with repeated cycling of pathogens from humans to the general environment.

Under this same model, the evolution of cross-kingdom pathogenicity can also be viewed as a secondary trait that arose following the acquisition of key factors by bacteria, which acted either as anti-feeding deterrents against potential animal predators, or functioned to ensure survival and persistence in the host and/or general environment [91]. These determinants may have targeted key defense pathways of animal predators, which would have included the innate immune system—the most basal and common defense pathway of many eukaryotes. This would have allowed pathogens carrying such determinants to exploit both humans/animals and plants by targeting a common pathway, enabling pathogen replication and dispersal [92]. Still, such pathogens would have had to rely on incidental host association and/or contact mediated by external processes, as the microbe would not have specific strategies for host entry or association. The maintenance of cross-kingdom pathogenicity would require only the selective pressure that initially resulted in the evolution of the virulence or defense factor.

A second model that can account for the evolution of the cross-kingdom pathogens has plant pathogenicity as the ancestral state [89], with established plant pathogens having evolved the ability to exploit animals (including humans) as alternative hosts. The simplest mode of transmission is direct contact with an epiphytically colonized or diseased plant by humans, whether by ingestion, scrapes or abrasions, or contact with the skin or other mucosal membranes, providing the bacteria with a direct route to a potential host. Several plant pathogens that cause opportunistic human infections are associated with commercially relevant crop plants. Species of *Pantoea*, for example, are found quite commonly on plants, including beets [15,20], maize [22], rice [23], and pineapple [21], and human infections occur frequently following abrasions caused by rose thorns and splinters, suggesting that these plant-associated strains can lead to human infection directly (Figure 1) [17,32-34]. Similarly, *Burkholderia* infects various plant species including onion, rice, sorghum and velvet beans [39,47,90], and may also have a direct route to humans. Indirect transfer to humans or even to the nosocomial environment may be mediated by other organisms like ants and flies, which have been implicated as carriers of many bacterial species [87,88], and may provide environmental isolates with a direct route to other environments and hosts.

Although there is evidence to support these models of evolution, it is difficult to establish evolutionary directionality, or the underlying selective pressures that lead to the evolution of cross-kingdom pathogenicity. The ability of human pathogens to exploit plants as alternative hosts is of particular significance from the perspective of host jumps and host-specific adaptation, since plants would ultimately serve as an extensive reservoir for clinically relevant bacteria. Some plant endophytes, which are organisms that live inside of plants often asymptomatically, have been shown to exhibit human pathogenic potential. Species like *Morganella morganii*, *Klebsiella pneumoniae*, *Pantoea agglomerans* and *Streptomyces* sp., have clinical relevance, but have been more closely investigated for their association with plants [93,94]. *M. morganii* is a phosphate solubilising bacterium [95], *K. pneumoniae* a nitrogen-fixing endophyte [96], *P. agglomerans* a gall-forming phytopathogen, and *Streptomyces* sp. a plant endophyte and plant pathogen [97-99]. Many of these animal-pathogenic endophytes have been shown to be latent plant pathogens, where after establishing a symbiotic mutualistic relationship with their plant host, the bacterium infects and causes severe disease within the plant host, possibly due to environmental changes [100]. It has also been observed that an organism that is an endophyte in one plant species may be pathogenic in a different plant species [101]. Yet, many human pathogens have been found to occur naturally in the rhizosphere [93], and it has been suggested that the mechanisms necessary for surviving in the rhizosphere are similar to those necessary for causing human infection [102]. Contributing to the maintenance of these crosskingdom pathogens may be anthropogenic factors. Certain species with human and plant pathogenic potential, such as *Pantoea* and *Burkholderia cepacia* are also commonly used as biocontrol agents against *Erwinia amylovora* and *Rhizoctonia solani*, respectively [103,104]. The extensive use of these bacteria as biocontrol agents on commercially relevant crops can lead to broad dispersal in the environment, increasing their populations dramatically and providing them with greater opportunity for interaction with other microbes. Horizontal gene transfer with other plant and human pathogenic bacteria in the environment can lead to extensive exchange of host-specific virulence factors or niche- specific determinants, creating rapidly-evolving populations that may exhibit oscillations between host-associative and free-living states.

## 5. Conclusions

Advances in molecular genetics coupled with the exploration of the pathogenic potential of seemingly dedicated pathogens is beginning to reveal that many phytopathogenic bacteria are capable of exploiting human hosts, and many human pathogens are capable of exploiting plant hosts. The constant cycling of pathogens from the general environment to human environments may help to maintain cross-kingdom pathogenicity, with plants serving as intermediate hosts or reservoirs for human pathogens. The ability of these cross-kingdom pathogens to maintain their population levels in a variety of environments likely increases their pan genome and evolutionary potential, ultimately making these pathogens significant from the perspective of emerging and remerging infectious diseases.
